# Prevalence and molecular characterization of group B streptococcus in pregnant women from hospitals in Ohangwena and Oshikoto regions of Namibia

**DOI:** 10.1186/s12866-021-02283-2

**Published:** 2021-08-05

**Authors:** Erastus Lafimana Haimbodi, Munyaradzi Mukesi, Sylvester Rodgers Moyo

**Affiliations:** Department of Health Sciences, Faculty of Health and Applied Sciences, Namibia University of Science and Technology, Windhoek, Namibia

**Keywords:** Group B streptococcus, Molecular characterization, Antimicrobial susceptibility

## Abstract

**Background:**

The main purpose of this study was to investigate the prevalence rate, antimicrobial susceptibility patterns and molecular characteristics of *Streptococcus agalactiae* isolated from pregnant women at 35 weeks of gestation and above, who attended antenatal screening at selected hospitals in Ohangwena and Oshikoto regions of Namibia.

**Results:**

Out of 210 women screened for Group B Streptococcus (GBS), 12 (5.7%) were colonised of which 25.0% were colonised rectovaginally, 58.0% vaginally and 17.0% rectally. No significant association was reported between GBS colonisation and maternal age, geographic location, marital status, education, employment, parity, still births and miscarriages (*P* values > 0.05). Antimicrobial susceptibility was reported at 100% for ampicillin, penicillin & ceftriaxone which are commonly used for empiric treatment of infection with GBS. Resistance to tetracycline was reported at 100%. Tetracycline resistance gene tet(M) was present in 88.9% of the isolates only and none of the isolates presented with tet(O). Polysaccharide capsular type Ia was found in 9(50%) and Ib was found in 1(5.5%) of the total isolates. The remaining isolates were not typeable using PCR.

**Conclusion:**

*Streptococcus agalactiae’s* positive rate was 5.7% among the pregnant women examined. Socio-demographic and obstetric factors had no influence on GBS colonisation (*P* values > 0.05). No resistance was reported to ampicillin, penicillin and ceftriaxone. No sensitivity was reported to tetracycline. Fifty percent of the isolates were capsular type Ia, 5.5% were type Ib and 44.4% were not typeable using PCR. The study provides crucial information for informing policy in screening of GBS in pregnant women.

## Background

*Streptococcus agalactiae* (*S. agalactiae*) is a gram-positive bacterium which belongs to the Lancefield Group B streptococcus (GBS). It is part of the normal flora of the gastrointestinal and female genitourinary tracts, and it is present in the genital tract of about 20–60% of pregnant women [1]. Nearly 20–40% of healthy women are colonised by GBS, and 50–70% of infants born to these mothers become infected with it [2].

Group B streptococcus is a leading cause of neonatal sepsis and meningitis worldwide [3]. It is widely implicated in maternal infections such as endometritis and chorioamnionitis as well as neonatal infections such as pneumonia, meningitis, sepsis and septicaemia [4]. Mortality of GBS in neonates is over 50% and is particularly high in preterm infants [3]. Vaginal colonisation by GBS during pregnancy is associated with premature delivery and still births [1].

The median prevalence of GBS in pregnant women in Africa was reported at 16% in 2009 [5]. The prevalence in Windhoek, Namibia was reported at 13.6% in 2016 [6]. Furthermore, the colonisation rate in other Southern African countries was reported at 31.6% in Zimbabwe in 2000 [7], 37% in South Africa in 2018 [8, 9], 21.2% in Malawi in 2010 [10] and 1.8% in Mozambique in 2008 [11].

Most studies done in Africa have shown a uniformity in sensitivity of GBS to penicillin and ampicillin which are the recommended drugs for prophylaxis against GBS. The Centres for Diseases Control and Prevention (CDC) recommends GBS screening in women at 35–37 weeks of gestation. Prophylaxis is given to pregnant women who are colonised by GBS, deliver infants with invasive GBS disease or are of unknown GBS status at delivery to prevent neonatal infection [12]. However, erythromycin & clindamycin are recommended for patients who are allergic to penicillin [12]. Gene based resistance in GBS is encoded by ermB, ermA, ermTR, mefA, mefE, aphA-3, aad-6, tet(M), tet(O), int-Tn and mreA [13]. Despite the availability of the CDC guidelines, Namibia has not adopted an approach to screen for GBS in pregnant women.

Group B streptococcus is classified into ten common capsular types based on capsular polysaccharide antigens on the surface of the organism. These capsular types are Ia, Ib, II, III, IV, V, VI, VII, VIII and IX [14, 15]. The most prominent capsular types in Southern Africa are type Ia, Ib, II, III and V [7, 8, 16–19]. The capsule is the main virulence factor for GBS. The study aimed to determine the prevalence and molecular characteristics of group B streptococcus isolated from pregnant women from selected rural settings of Namibia.

## Results

### Prevalence of group B streptococcus

A total of 12 out of the 210 participants, (5.7%) tested positive for GBS colonisation, of which 7 (3.3%) tested positive with vaginal swabs and 2 (1.0%) tested positive with rectal swabs. A total of 3 (1.4%) out of 210 participants tested positive with both rectal and vaginal swabs.

This study assessed the influence of different socio-demographic characteristics, age and obstetric factors on the rate of GBS colonisation in pregnant women screened.

The age of pregnant women screened in this study ranged from 16 to 49 years with mean age of 29 years and standard deviation of ±7. Out of the total 210 pregnant women, 174 were living in rural, 9 in semi-urban and 27 in urban areas. One hundred and sixty (160) women were not married, while 50 were married. Those with qualifications below matric were 156, those with matric qualifications were 51 and those with tertiary qualifications were only 3. Unemployed women were 168 and employed women were 42.

Out of the twelve colonised women, 75.0% was women aged between 20 and 39 years and 25.0% was women aged 40 years and above (*P* value 0.209). Majority of colonised women were rural based women (83.3%) than urban living women (16.7%) (*P* value 0.708). Unmarried women colonised by GBS were 75.0 and 25.0% of colonised women were married (*P* value 0.921).

Majority of colonised women were those with educational level below matric (58.3%) than those with matric qualifications (41.7%) (*P* value 0.333). The same scenario was found in terms of employment status, whereby 66.7% of unemployed women were colonised and only 33.3% of colonised women were employed (*P* value 0.234).

Majority of GBS colonised were unmarried, unemployed and rural based women aged between 20 and 39 years who never matriculated. Table 1 illustrates the rates of GBS colonisation based on various socio-demographic factors.

**Table 1:** Association between socio-demographic factors and GBS colonisation

Characteristic	Category	GBS non-colonised frequency (***n*** = 198)	GBS colonised frequency (***n*** = 12)	***P*** value
Age in years	Below 20	12 (6.1%)	0 (0.0%)	0.209
	20–39	166 (83.8%)	9 (75.0%)	
	40 and above	20 (10.1%)	3 (25.0%)	
Geographic location	Rural	164 (82.8%)	10 (83.3%)	0.708
	Semi-urban	9 (4.5%)	0 (0.0%)	
	Urban	25 (12.6%)	2 (16.7%)	
Marital status	Not married	151 (76.3%)	9 (75.0%)	0.921
	Married	47 (23.7%)	3 (25.0%)	
Educational level	Below matric	149 (75.3%)	7 (58.3%)	0.333
	Matric	46 (23.2%)	5 (41.7%)	
	Tertiary	3 (1.5%)	0 (0.0%)	
Employment status	Unemployed	160 (80.8%)	8 (66.7%)	0.234
	Employed	38 (19.2%)	4 (33.3%)	

Among all GBS colonised women, women with three or more successful pregnancies were 50.0%, while those with either one or two were 33.0% and those without any previous pregnancy were 16.7% (*P* value 0.659). Carriage of GBS was only shown in women without previous history of still births (*P* value 0.613). GBS colonised women without any history of miscarriages were 83.3% while those with a history of either 1 or 2 miscarriages were 16.7% (*P* value 0.471). Table 2 illustrates the association between obstetric factors and GBS colonisation.

**Table 2:** Association of obstetric factors and GBS colonisation among pregnant women

Characteristic	Category	GBS non-colonised frequency (***n*** = 198)	GBS colonised frequency (***n*** = 12)	***P*** value
Parity	0	43 (21.7%)	2 (16.7%)	0.659
	1–2	82 (41.4%)	4 (33.3%)	
	≥3	73 (36.9%)	6 (50.0%)	
Still births	0	183 (92.4%)	12 (100%)	0.613
	1–2	13 (6.6%)	0 (0.0%)	
	≥3	2 (1.0%)	0 (0.0%)	
Miscarriages	0	178 (89.9%)	10 (83.3%)	0.471
	1–2	20 (10.1%)	2 (16.7%)	
	≥3	0 (0.0%)	0 (0.0%)	

The culture of rectal and lower vaginal swabs by both direct BCNA agar and Todd-Hewitt-BCNA agar yielded a total of 18 CAMP positive GBS isolates recovered from 12 women. These isolates were confirmed as *S. agalactiae* using real time PCR, in which the target gene *scp*B for C5a peptidase gene was amplified. Following gel electrophoresis, DNA amplicons approximately 255 base pairs on the gel were identified as *S. agalactiae*. No sequencing was performed in this study.

### Antimicrobial susceptibility patterns

All 18 GBS isolates obtained from this study were tested individually for antimicrobial susceptibility to each of the antibiotics used, and all showed 100% susceptibility to penicillin, ampicillin, ceftriaxone, clindamycin, erythromycin, vancomycin, linezolid and chloramphenicol. All isolates revealed 100% resistance to tetracycline. Intermediary sensitivity and inducible clindamycin resistance were reported for any organism. The frequencies of antimicrobial susceptibility patterns are illustrated in Table 3.

**Table 3:** Antimicrobial susceptibility patterns of GBS isolates to commonly used drugs

Antimicrobial agent	Percentage of GBS antibiotics sensitive, intermediate and resistant					
	Sensitive (S)		Intermediate (I)		Resistant (R)	
	Frequency	Percentage	Frequency	Percentage	Frequency	Percentage
Penicillin	18	100%	0	0%	0	0%
Ampicillin	18	100%	0	0%	0	0%
Ceftriaxone	18	100%	0	0%	0	0%
Erythromycin	18	100%	0	0%	0	0%
Clindamycin	18	100%	0	0%	0	0%
Vancomycin	18	100%	0	0%	0	0%
Tetracycline	0	0%	0	0%	18	100%
Linezolid	18	100%	0	0%	0	0%
Chloramphenicol	18	100%	0	0%	0	0%

Ceftriaxone was performed using Kirby-Bauer disc diffusion method for all isolates.

Consistent minimum inhibitory concentrations (MICs) were observed in most antibiotics. These include ampicillin (≤0.25 μg/L), penicillin (≤0.12 μg/L), clindamycin (≤0.25 μg/L), chloramphenicol (≤10 μg/L) and tetracycline (≥16 μg/L). The highest MIC value reported in erythromycin was 0.5 μg/L and the lowest was ≤0.25 μg/, which were all considered sensitive.

The highest MIC value reported for vancomycin was 4 μg/L and the lowest was ≤0.5 μg/L, which were all considered sensitive. Linezolid was reported sensitive with MICs ranging from 1 μg/L to 2 μg/L. The smallest inhibitory zone reported for ceftriaxone was 25 mm and the biggest was 33 mm which were all considered as sensitive.

### Gene based resistance of GBS isolates

All isolates were tested for the presence of tetracycline resistance genes i.e. *tet*(M) and *tet*(O) with multiplex PCR, using primers described in previous studies [13, 20]. In total, 16 isolates possessed *tet*(M) whereas *tet*(O) was found in none of the isolates, although they were all phenotypically resistant to tetracycline. Amplicons for *tet*(M) have 347 base pairs and for *tet*(O) have 548 base pairs [20].

### Polysaccharide capsular typing

Following the amplification and electrophoresis, 9 isolates presented with amplicons of 521 base pairs and 1 isolate with an amplicon sized 770 base pairs. An amplicon of 521 base pairs is associated with capsular polysaccharide Ia whereas 770 base pairs is associated with capsular type Ib. Polysaccharide capsular type Ia was found in 9 (50%) of isolates and Ib was found in 1 (5.5%) of the total isolates. The remaining eight (44.4%) isolates could not be classified using PCR. Polysaccharide capsular type IX was not assessed in this study.

## Discussion

The prevalence of GBS among pregnant women found in this study (5.7%) was lower compared to what was reported in a similar study conducted in Windhoek, Namibia in 2016, whereby the prevalence of GBS colonisation was reported at 13.6% [6]. Different recruitment centres were included in the current study, which comprise of a different population characteristic from the study by Engelbrecht et al., 2016 [6]. The prevalence in this current study is lower than the 31.6% found in Zimbabwe in 2000 [7], 30.9% in South Africa in 2012 [9, 17], 37% in South Africa in 2019 [8] and 21.2% in Malawi in 2010 [10].

The findings of this current study are similar to those reported in a Mozambican study, whereby a colonisation rate of 1.8% was obtained [11]. However, in the Mozambican study swabs were inoculated in Todd-Hewitt broth with only gentamycin (8 μg/ml of agar) and the broth was sub-cultured onto human blood agar. The use of gentamycin alone without nalidixic acid can cause overgrowth of enterobacteriaceae organisms over GBS, hence reducing the growth of GBS. Furthermore, human blood used in preparation of blood agar might have contained inhibitory substances against the growth of GBS [11].

In the current study, 10 (83.3%) women colonised with GBS were rural based while 2 (16.7%) were urban based. This difference was however not statistically significant (*P* value 0.708). In a similar study conducted in Dr. George Mukhari hospital, South Africa, more of the colonised women were dwelling in the urban areas [9]. This finding was however also not statistically significant (*P* value 0.08). It was observed in this study that majority (9) of GBS colonised women were not married (75.0%), compared to married women (3). There was however no significant statistical association between GBS colonisation and marital status (*P* value 0.921). This agrees with the study conducted at Dr. George Mukhari hospital, South Africa, which reported that the majority of colonised women were single, divorced or widowed followed by cohabiting women (*P* value 0.56) [9]. In another study conducted in Windhoek by Engelbrecht et al., 2016, more single women were colonised compared to married women (*P* value 0.315) [6]. Both findings were not statistically significant.

Group B streptococcus colonisation did not appear to be linked to obstetric factors such as history of miscarriages and still births, as women with no history of still births (12) and miscarriages (10) were largely colonised (100 and 83.3% respectively). These findings did not have any statistical significance (*P* value 0.613 and 0.471 respectively). However, GBS colonisation had shown to increase with parity, 50.0% in women with parity exceeding two, 33.3% in women with one or two previous deliveries and 16.7% in women without previous deliveries. This was however not statistically significant (*P*. value 0.659). In the Saudi Arabian study conducted at Makkah, a significant statistical association was found between parity and GBS colonisation [21].

Similarly, GBS colonisation did not appear to be linked to history of miscarriages and still births in the South African study, as the carriage rate was greater in women who had no history of miscarriages and still births compared to the opposite. These were equally not statistically significant findings (*P* value 0.33 and 0.24 respectively) [9]. In contrast to the findings of this current study, a study by Monyama et al., 2016 [9], in South Africa reported that women with multiple deliveries exceeding two were less affected by GBS compared to women who had one to two deliveries and those with zero parity. However, there was no statistical significance (*P* value 0.24) [9].

In the current study, GBS showed sensitivity to penicillin, ampicillin, ceftriaxone, vancomycin, chloramphenicol and linezolid by 100%. This correlates with the findings of the study done in Windhoek, where 100% susceptibility was reported to penicillin, ampicillin, ceftriaxone, vancomycin and linezolid. All isolates were found to be sensitive to clindamycin and erythromycin by 100%. This does not correlate with findings of the Windhoek study, whereby 88.9% sensitivity and 11.1% resistance was reported for erythromycin, while for clindamycin 72.6% sensitivity, 18.8% intermediate sensitivity and 8.5% resistance was reported [6].

Since only 18 GBS isolates were obtained from the current study, 100% susceptibility to clindamycin and erythromycin might not be a true reflection of the antimicrobial susceptibility patterns of GBS in the two regions, as a bigger number of isolates might have reflected a different picture. Furthermore, this current study revealed a 100% resistance to tetracycline, while the previous study has revealed 94.9% resistance to tetracycline and 5.1% susceptibility thereof. This difference may be attributable to the differences in the locations of the two studies and also different sample sizes. High levels of resistance to tetracycline by GBS is an indication of growing resistance to commonly used antibiotics especially those used in both humans and animals [6–11].

The findings of our study are in correlation with another study conducted in Ayder referral hospital and Mekelle health centre, Mekelle, Northern Ethiopia, whereby GBS was reportedly 100% susceptible to penicillin, ampicillin, erythromycin, and vancomycin [1]. However, in the same study, intermediate GBS susceptibility was reported to chloramphenicol by 42.1% and ceftriaxone by 26.3%, which opposes the findings of our study [1].

On the contrary, our study findings are different from the findings of a similar study conducted in Ghandi memorial and Tikur Anbessa, Addis Ababa, Ethiopia, where GBS was found to be 55% susceptible to penicillin, 91% sensitive to ampicillin and all isolates except one were susceptible to erythromycin [22]. Increased resistance to penicillin in this area was attributable to a wide non-prescription use of penicillin due to weak drug control mechanisms [22].

Another study conducted in Gabon, Central Africa to determine capsular type distribution and antimicrobial susceptibility of GBS in pregnant women found that all GBS isolates were susceptible to clindamycin, linezolid, vancomycin and benzyl penicillin which correlates with the findings of our study [16]. Fourteen isolates were however found intermediary susceptible to erythromycin, which contrasts the findings of our study. However, no inducible clindamycin resistance was found in the Gabon study, which draws a parallel between the two studies [16].

In the neighbouring South Africa, a study conducted to assess the antimicrobial resistance of *S. agalactiae* isolated from pregnant women in Garankuwa found that all strains were 100% susceptible to penicillin, ampicillin and vancomycin, which is similarly demonstrated in our study [8]. The same study however found some strains to be resistant to erythromycin, clindamycin, tetracycline and chloramphenicol by 21.1, 17.2, 94.5 and 24.9% respectively, which distinguishes the two study findings [8]. In a comparable study conducted in Soweto, South Africa to determine the risk of GBS sepsis in infants exposed to HIV, all isolates were penicillin sensitive, and macrolide resistance was observed in 5.5% [23].

Generally, GBS has shown uniform sensitivity to penicillin, ampicillin, vancomycin, ceftriaxone and linezolid across many settings. Resistance to tetracycline was reported at 100% in our study compared to many previous studies, even though they also reported quite high percentages of resistance to tetracycline. Resistance to erythromycin and clindamycin was however not reported in our study, as opposed to other studies. These differences may be attributed to variations in capsular types common in different locations. In additions, fewer GBS isolates were obtained in the current study as compared to other studies, hence the differences in percentages of antimicrobial susceptibility.

None of the isolates phenotypically resistant to tetracycline presented with *tet*(O) gene, which confers resistance against tetracycline in addition to *tet*(M) gene [13]. A related study which was conducted in Windhoek has found *tet*(M) in 114/117 GBS isolates and of these, 111 were tetracycline resistant as interpreted by the Vitek 2 [6]. Based on that study, six of the isolates that possessed *tet*(M) did not appear to be phenotypically resistant to tetracycline [6]. This does not concur with our findings, as all isolates presenting with *tet*(M) were phenotypically resistant to tetracycline in our study. Only tetracycline resistance genes were inspected in our study, as all isolates from this study have only shown phenotypical resistance to tetracycline.

Our study found capsular type Ia to be common among the pregnant women screened. A similar study conducted to determine the antigenic distribution of *S. agalactiae* isolates from pregnant women at Garankuwa hospital in South Africa revealed that capsular type III was the most common capsular type (29.7%), followed by Ia (25.6%), II (15.6%) IV (8.6%), V (10.9%) and Ib (8.6%) [17]. These imply that the prominent capsular types associated with this population were III, Ia and II. Differences in geographical locations of the two studies may attribute to the differences in capsular types found in the two studies. Non-typable strains reported in this study could have been a result of capsular type IX which was not assessed. A combination of both serology and molecular analysis may also have improved classification of the non-typables.

Another South African study conducted on pregnant mothers attending prenatal clinics in Soweto concluded that capsular type III was more associated with persistent colonisation throughout the study (29%) than Ia (18%) and V (6%) [18]. In a Zimbabwean study, the following capsular types were identified as follows: la, lb., II, III and V at 15.7, 11.6, 8.3, 38.8 and 24.0% respectively [19], which corresponds with our findings.

GBS colonises pregnant women presenting the risk of vertical transmission to the baby during the process of giving birth. Although the prevalence of GBS was low in this current study, it provides further information that some women are colonised with GBS at the late stages of pregnancy and are at risk of passing it on to the babies. Antimicrobial resistance was low to commonly used antibiotics except tetracycline in this study. However, there is need for continuous monitoring of antibiotic resistance as studies in other geographic locations have reported varying degrees of resistance by GBS to antibiotics. Although GBS is not routinely screened in pregnant women in Namibia, the results of this study are important for informing future policy considerations on its screening and management among pregnant women in the country and for empirical treatment of those colonised with GBS.

## Conclusions

In the current study, the prevalence of GBS colonisation in women who attended antenatal screening at Okongo, Eenhana and Onandjokwe state hospitals was 5.7%. Twelve women were colonised in total and of these, 25.0% carried GBS in their lower vagina and rectum, 58.0% in the lower vagina only and 17.0% in the rectum only. Our study findings have revealed that there was no significant statistical association between GBS colonisation and age, habitat, marital status, educational level and employment status. Similarly, there was also no statistically significant association between GBS colonisation and parity, history of still births and miscarriages.

Antimicrobial susceptibility was reported at 100% sensitive for ampicillin, penicillin, ceftriaxone, clindamycin, erythromycin, vancomycin, linezolid and chloramphenicol. Resistance to tetracycline was reported at 100%. However, 100% susceptibility to erythromycin and clindamycin may be due to a smaller number of GBS isolates obtained from this study. The tetracycline resistance gene *tet*(M) was present in 88.9% of isolates and 11.1% had none, regardless of being phenotypically resistant to tetracycline. None of the isolates possessed detectable *tet*(O) resistance gene.

Polysaccharide capsular type Ia was found in 9 (50%) of isolates and Ib was found in 1 (5.5%) of the total isolates. The remaining 8 (44.4%) isolates could not be classified using PCR.

## Methods

### Study design

This was a descriptive cross-sectional study which gathered descriptive data such as socio-economic, demographic characteristics and obstetric factors directly from participants using a questionnaire.

### Study population

The population comprised of pregnant women between 35 and 37 weeks gestation, who attended antenatal clinics at Onandjokwe, Eenhana and Okongo state hospitals between May and September 2018. Women who had taken antibiotics 2 weeks prior to the study, as well as those aged below 16 years were excluded from the study.

### Sample size

The sample size was calculated based on the prevalence rate of GBS colonisation in pregnant women found in Windhoek, which was 13.6% [6], using the following formula adopted from a prior study [1]. The expected sample size was 181 participants (*n* = 181 participants).

A total of 210 subjects were screened, 98 from Onandjokwe hospital, 93 from Eenhana hospital and 19 from Okongo hospital.

### Specimen collection

A convenience sampling technique was used in this study and samples were collected consecutively. A lower vaginal and a rectal swab were collected from each participant by qualified clinicians using sterile cotton-tipped swabs. A lower vaginal swab was collected by inserting and brushing a sterile swab 2 cm into the vagina, periurethral area and labia. A rectal swab was collected by inserting a sterile swab 1 cm into the anus, rubbing the wall of the anal canal as described before [12]. The swabs were inserted into Amie’s transport medium for preservation of bacteria. Each specimen was labelled with a unique identification. Specimens were transported at 2–8 °C to the laboratory within an hour from collection. A cold chain protocol was observed during specimen transport and samples were cultured within an hour after reaching the laboratory.

### Specimen culturing

Each swab was inoculated directly onto 5% Sheep blood agar with colistin and nalidixic acid (BCNA), followed by inoculation into Todd-Hewitt broth (TH) supplemented with colistin and nalidixic acid. The culture plates and broths were incubated at 37 °C in CO_2_ for 18–24 h. After incubation, BCNA culture plates were examined for presence of large, whitish-grey and translucent colonies with a narrow zone of β-haemolysis while the Todd-Hewitt broths were subcultured onto BCNA agar. Subcultured BCNA plates were incubated at 37 °C in CO_2_ for 18–24 h and examined for presumptive GBS colonies. The Columbia CNA agar, Todd Hewitt broth, colistin and nalidixic acid antibiotics were obtained from Rochelle Chemicals and Laboratory Equipment, Johannesburg, South Africa.

### Isolation and identification of GBS

Single colonies that presented with a narrow or large zone of β-haemolysis on the BCNA or TH-BCNA were subjected to the CAMP test for presumptive identification. *S. agalactiae* ATCC 12403 was used as a positive control and *Streptococcus pyogenes* ATCC 1244 as negative control. A known β-haemolytic toxin producing *Staphylococcus aureus* clinical isolate was used in this test. Presumptive GBS isolates were tested again using Vitek 2 before they were confirmed as GBS using molecular techniques by amplifying the *scp*B gene [24].

### DNA extraction

DNA of positive and negative controls (ATCC 12403 and ATCC 1244 respectively) and test isolates was extracted using the boiling method as described in a prior study [4]. Pure cultures of presumptive isolates were harvested from nutrient agar plates and emulsified in 200 μL of DNA free water in respective Eppendorf tubes using sterile plastic loops, followed heating at 100 °C on the heating block for 15 min. Eppendorf tubes were centrifuged at 8000 rpm (rpm) for 10 min. The supernatant containing DNA was transferred to a clean labelled tube, and stored at − 20 °C before use.

### PCR for identification

A primer pair from Inqaba Biotechnical Industries (Pty) Ltd. (Pretoria, South Africa) 5′–ACAACGGAAGGCGCTACTGTTC–3′ (forward primer) and 5′–ACCTGGTGTTTGACCTGAACTA–3′ (reverse primer) which targeted the *scp*B gene was used for confirmation of GBS [20]. PCR reaction mixture was prepared as follows: 12 μl One *Taq®* Quick load® 2X master mix with standard buffer, 10.5 μl nuclease free water, 1 μl of forward and reverse primer, and 5 μl DNA template, giving a final volume of 29.5 μl. The PCR conditions were as follows: 1 cycle at 94 °C for 4 min (initial denaturation), 35 cycles at 93 °C for 1 min (denaturation), 57.6 °C for 1 min (annealing), 72 °C for 1 min (elongation), 1 cycle at 72 °C for 7 min (further elongation) and 4 °C dwelling temperature using a BioRad thermal cycler (Johannesburg, South Africa).

The amplicons were electrophoresed on a 2% agarose gel electrophoresis with 1xTris-Acetate EDTA (TAE) buffer, at 110 V for 45 min. A 1000 base pair (bp) DNA ladder, positive and negative control were included. The gel was visualised under the SYN GENE BIO IMAGING ultra violet system (Cambridge, United Kingdom). The sizes of amplicons were determined using the DNA molecular weight size standard.

### Antimicrobial sensitivity testing

Antimicrobial sensitivity testing for sixteen confirmed isolates was performed using the Vitek 2 system (bioMerieux, Midrand, South Africa). This method employed gram positive antimicrobial sensitivity cards (AST-ST01). This card tested for penicillin, ampicillin, erythromycin, clindamycin, vancomycin, tetracycline, linezolid & chloramphenicol. Ceftriaxone was tested using the Kirby-Bauer disk diffusion method. Two isolates were tested using the Kirby-Bauer disc diffusion method against all antibiotics. Minimum inhibitory concentration (MIC) breakpoint results and disc zones were interpreted as stipulated by the Clinical Laboratory Standards Institute [25].

### Multiplex PCR for gene-based resistance testing

Specific oligonucleotide primers (Table 4) from Inqaba Biotechnical Industries (Pty) Ltd. (Pretoria, South Africa) were used to screen for genotypic resistance [13, 26]. The reaction mixture was prepared as follows: 12 μl One *Taq®* Quick load® 2X master mix with standard buffer, 10.5 μl nuclease free water, 1 μl of all forward and reverse primers, and 5 μl DNA template, giving a final volume of 31.5 μl. The multiplex PCR conditions were as follows: 1 cycle at 94 °C for 4 min (initial denaturation), 35 cycles at 93 °C for 1 min (denaturation), 57.6 °C for 1 min (annealing), 72 °C for 1 min (elongation), 1 cycle at 72 °C for 7 min (further elongation) and 4 °C dwelling temperature using a BioRad thermal cycler (Johannesburg, South Africa). The amplicons were electrophoresed on a 2% agarose gel electrophoresis with 1xTris-Acetate EDTA (TAE) buffer, at 110 V for 45 min. A 1000 base pair (bp) DNA ladder, positive and negative control were included. The gel was visualised under the SYN GENE BIO IMAGING ultra violet system (Cambridge, United Kingdom). The sizes of amplicons were determined using the DNA molecular weight size standard.

**Table 4:** Oligonucleotide primers used in multiplex PCR for gene based resistance testing

Primer	Target	Primer sequence 5′ – 3’	Size
*tet*mS – F	*tet*(M)	GTCTTGCATATATACGCCTTTATAGTGGAGTACTACATTTACGAG	374 bp
*tet*mA – R	*tet*(M)	CCACGTAATATCGTAGAAGCGGATCACTATCTGAG	
*tet*oS – F	*tet*(O)	CGTATATATAGCGGAACATTGCATTTGAGGG	548 bp
*tet*oA – R	*tet*(O)	CGGCTCTATGGACAACCCGACAGAAG	

### PCR for polysaccharide capsular typing

Oligonucleotide primer pairs (Table 5) from Inqaba Biotechnical Industries (Pty) Ltd. (Pretoria, South Africa) were used in capsular polysaccharide typing, in three different categories, i.e. type Ia – III, IV – VII and VIII [21]. The PCR reaction mixture was prepared as follows: 12 μl One *Taq®* Quick load® 2X master mix with standard buffer, 10.5 μl nuclease free water, 1 μl of all forward and reverse primers, and 5 μl DNA template, giving a final volume of 35.5 μl for type Ia – III & IV – VII, and a final volume of 29.5 μl for type VIII categories.

**Table 5:** Oligonucleotide Primers for polysaccharide capsular typing

Primer	Target	Primer sequence	Size bp
Ia-F	*cps1aH*	5′ – GGTCAGACTGGATTAATGGTATGC – 3′	521 & 1826 bp
Ia-R	*cps1Ah*	5′ – GTAGAAATAGCCTATATACGTTGAATGC – 3′	
Ib-F	*cps1bJ*	5′ – TAAACGAGAATGGAATATCACAAACC – 3′	770 bp
Ib-R	*cpsIbK*	5′ – GAATTAACTTCAATCCCTAAACAATATCG – 3’	
II-F	*cps2K*	5′ – GCTTCAGTAAGTATTGTAAGACGATAG – 3’	397 bp
II-R	*cps2K*	5′ – TTCTCTAGGAAATCAAATAATTCTATAGGG – 3’	
III-F	*cps1a/2/3I*	5′ – TCCGTACTACAACAGACTCATCC – 3’	1826 bp
III-R	*cps1a/2/3 J*	5′ – AGTAACCGTCCATACATTCTATAAGC – 3’	
IV-F	*cps4N*	5′ – GGTGGTAATCCTAAGAGTGAACTGT – 3’	578 bp
IV-R	*cps4N*	5′ – CCTCCCCAATTTCGTCCATAATGGT – 3’	
V-F	*cps5O*	5′ – GAGGCCAATCAGTTGCACGTAA – 3’	701 bp
V-R	*cps5O*	5′ – AACCTTCTCCTTCACACTAATCCT – 3’	
VI-F	*cps6I*	5′ – GGACTTGAGATGGCAGAAGGTGAA – 3’	487 bp
VI-R	*cps6I*	5′ – CTGTCGGACTATCCTGATGAATCTC – 3’	
VII-F	*cps7M*	5′ – CCTGGAGAGAACAATGTCCAGAT – 3’	371 bp
VII-R	*cps7M*	5′ – GCTGGTCGTGATTTCTACACA – 3’	
VIII-F	*cps8J*	5′ – AGGTCAACCACTATATAGCGA – 3’	282 bp
VIII-R	*cps8J*	5′ – TCTTCAAATTCCGCTGACTT – 3’	

The thermocycling conditions were as follows: 1 cycle at 94 °C for 4 min (initial denaturation), 35 cycles at 93 °C for 1 min (denaturation), 58 °C for 1 min (annealing) (for type Ia – III), 59 °C (for type IV – VII) & 56 °C (for type VIII), 72 °C for 1 min (elongation), 1 cycle at 72 °C for 7 min (further elongation) and 4 °C dwelling temperature using a BioRad thermal cycler (Johannesburg, South Africa). The amplicons were electrophoresed on a 2% agarose gel electrophoresis with 1xTris-Acetate EDTA (TAE) buffer, at 110 V for 45 min. A 1000 base pair (bp) DNA ladder, positive and negative control were included. The gel was visualised under the SYN GENE BIO IMAGING ultra violet system (Cambridge, United Kingdom). The sizes of amplicons were determined using the DNA molecular weight size standard.

### Data analyses

Data was analysed using the Statistical Package for Social Sciences version 22. The Pearson’s Chi square was used to test for relationships between categorical variables. Descriptive statistics were used to summarise the frequencies and they were presented as cross tabulations. A *P* value less than 0.05 represented statistical significance.

## Data Availability

The datasets used and/or analysed during the current study are available from the corresponding author on reasonable request.

## Abbreviations

A: Adenine
ATCC: American Type Culture Collection
BBB: Blood Brain Barrier
BCNA: Columbia Sheep Blood with Colistin and Nalidixic acid
BibA: GBS immunogenic bacterial adhesion
C: Cytosine
CAMP: Christie, Atkins, Munch and Petersen test
CDC: Centres for Diseases Control and Prevention
CLSI: Clinical Laboratory Standards Institute
DNA: Deoxy Ribonucleic Acid
EOD: Early Onset Disease
G: Guanine
GBS: Group B *Streptococcus*
HIV: Human Immuno-Deficiency Virus
IAP: Intrapartum Antibiotic Prophylaxis
Lmb: Laminin binding protein
LOD: Late Onset Disease
LVS: Lower Vaginal Swab
MIC: Minimum Inhibitory Concentration
NIP: Namibia Institute of Pathology
NUST: Namibia University of Science and Technology
PCR: Polymerase Chain Reaction
rRNA: Ribosomal Ribonucleic Acid
*S. agalactiae*: *Streptococcus agalactiae*
*S. aureus*: *Staphylococcus aureus*
SBA: Sheep Blood Agar
*scp*B: Group B streptococcal C5a peptidase
SfbA: Streptococcal fibronectin binding protein
SPSS: Statistical Package for Social Sciences
*S. pyogenes*: *Streptococcus pyogenes*
T: Thymine
TAE: Tris-Acetate EDTA buffer
Taq: Polymerase enzyme produced by *Thermophilus aquaticus*
TH: Todd Hewitt
